# Radiation induced preparation of polymer blends based on poly(vinyl alcohol/xanthan) polymers as gas sensors for food packing

**DOI:** 10.1038/s41598-025-27912-y

**Published:** 2025-11-29

**Authors:** Mohamed Shoaib, Reham Helal, Yasser K. Abdel-Monem, Farag A. Ali, Hussein Oraby, Magdy M. H. Senna

**Affiliations:** 1 Department, of Chemistry, Faculty of Science, Menoufiya University, Shebin El-Kom, Egypt; 2 Department of Chemical Engineering, Military Technical College, Cairo, Egypt; 3 Radiation Chemistry Department, National Center for Radiation Research and Technology, Egyptian Atomic Energy Authority, Cairo, Egypt

**Keywords:** Poly(vinyl alcohol), Xanthan, EB irradiation, Gas sensors, Food packing

## Abstract

Poly vinyl alcohol/xanthan gum (PVA/XG) blend films were fabricated via solution casting and then exposed to various doses of electron beam (EB) irradiation. The results indicated that the EB treatment has been significantly modified the structural and mechanical properties of the blends compared to pristine PVA. Fourier-transform infrared (FTIR) spectroscopy indicated the formation of hydrogen bonding and enhanced the interfacial adhesion and compatibility between the components. These findings were further supported by scanning electron microscopy (SEM) and tensile testing. Notably, the irradiated PVA/XG films exhibited rapid color changes in response to fish spoilage, highlighting their potential as reliable, antimicrobial-resistant freshness indicators for food packaging applications.

## Introduction

Hydrogels are three-dimensional polymeric networks capable of absorbing and retaining substantial amounts of water while maintaining their structural integrity. Among them, polyvinyl alcohol (PVA) has attracted significant attention due to its biocompatibility, flexibility, transparency, and chemical resistance to oils, solvents, and oxygen permeation^1–4^. However, PVA hydrogels are limited by their relatively low mechanical strength and slow swelling response^5^. To overcome these limitations, radiation-induced cross-linking has been widely investigated as a clean and effective approach to enhance the structural stability and functional performance of hydrogels. The incorporation of hydrophilic polysaccharides such as xanthan gum (XG) offers an effective strategy for improving the physicochemical properties of PVA hydrogels^6^. XG is a natural macromolecule rich in hydroxyl and carboxyl groups, which facilitates hydrogen bonding and enhances polymer compatibility, morphology, and water affinity^7–11^. Previous studies have reported the preparation of PVA/XG hydrogels using chemical cross-linkers such as glutaraldehyde^10^; however, the toxicity of chemical cross-linkers restricts their application in food-related and environmentally friendly materials. Electron beam (EB) irradiation provides a non-toxic, solvent-free method to induce cross-linking, improve interfacial adhesion, and tailor the structural and mechanical properties of polymer blends^12^. Growing environmental concerns over the accumulation of non-biodegradable plastic waste have accelerated research into biodegradable and sustainable packaging materials. Biopolymers such as starch, cellulose, chitosan, and gums are being explored for smart and active packaging applications^13^. Intelligent packaging systems capable of monitoring food freshness are particularly valuable, as they can detect spoilage by responding to volatile compounds released during decomposition. For illustration, ammonia generated during fish spoilage can trigger a visible color change in pH-sensitive indicators, enabling real-time freshness monitoring^14^. Recent research has increasingly focused on PVA/XG blends for food packaging, emphasizing improvements in mechanical strength, barrier properties, and biodegradability. For instance, films of PVA-XG cast without irradiation have been shown to significantly decrease moisture content, water solubility, and water vapor transmission while maintaining transparency and achieving comparable tensile strength to commercial plastics^15^. Another study reinforced chitosan-XG blends with ZnO nanoparticles to improve tensile strength and overall degradability^16^. More recently, enhancements of Xylan/PVA films via nano-ZnO have demonstrated sustainable packaging performance with improved physico-mechanical properties^17^. Also, PVA/gellan gum bioplastics incorporating guava and chickpea extracts revealed enhanced thermal and mechanical behavior and good compatibility for food packaging applications^18^. In contrast, the present work introduces electron beam (EB) irradiation as a green, additive-free modification route for producing PVA/XG blend films with enhanced mechanical properties and sensitivity to volatile amines released during food spoilage. Therefore, the novelty of this study lies in integrating EB irradiation with a biodegradable PVA/XG hydrogel network to generate an environmentally friendly, smart packaging material capable of freshness detection. In this study, PVA/XG blend films containing a pH indicator were prepared via solution casting and cross-linked using EB irradiation. The films were characterized by Fourier-transform infrared spectroscopy (FT/IR), scanning electron microscopy (SEM), X-ray diffraction (XRD), and swelling studies to assess their structural and mechanical properties. Furthermore, their ability to detect ammonia released from fish spoilage and their resistance to microbial degradation were evaluated, demonstrating their potential as eco-friendly, intelligent food packaging materials.

## Experimental

### Materials

Poly(vinyl alcohol) (PVA) with molecular weight of 130.000 g/mol was purchased from Loba Chemie, Mumbai, India. Xanthan Gum (XG) in the powder form with molecular weight of 1.16 × 106 g/mol was purchased from (Bhiwadi, Rajasten, India). Bromo Cresol purple (Powder dye) with molecular weight of 540 g/mol.

### Preparation of PVA/XG blends

Poly(vinyl alcohol) (PVA, Mw ≈ 89,000–98,000, 99% hydrolyzed) and xanthan gum (XG, food grade, Mw ≈ 2 × 10⁶) were used for blend preparation via the solution casting technique. The PVA powder was first dissolved in distilled water (10 wt %) at 80 °C under constant magnetic stirring until a clear viscous solution was obtained. Separately, xanthan gum was dispersed in distilled water (1 wt%) at room temperature and stirred for 1 h to ensure complete hydration. After complete dissolution, the two solutions were mixed at various weight ratios to produce films with different compositions: 100/0, 90/10, 80/20, and 50/50 (PVA/XG, w/w). The total polymer mass in each mixture was kept constant at 10 g. The blended solutions were stirred at 60 °C for 2 h to ensure uniform distribution of the polysaccharide phase within the PVA matrix. The homogeneous mixtures were then degassed and cast into clean glass Petri dishes, followed by drying at ambient temperature (25 ± 2 °C) for 48 h to form transparent films. The dried films were carefully peeled off and conditioned at 50% relative humidity before further analysis. Each composition was subsequently subjected to electron beam (EB) irradiation at doses of 5, 10, 15, and 25 kGy using a 3 MeV accelerator (National Center for Radiation Research and Technology, Egypt). The irradiated samples were stored in sealed polyethylene bags for characterization and testing.

### Electron beam irradiation

The prepared PVA/XG blend films were subjected to electron beam (EB) irradiation using a 3 MeV accelerator at the National Center for Radiation Research and Technology (NCRRT), Cairo, Egypt. The irradiation was performed at doses of 5, 10, 15, and 25 kGy under ambient conditions in air. After irradiation, the films were air-dried at room temperature to obtain uniform hydrogel structures suitable for further characterization.

### Characterization and analysis

#### Swelling behavior

The samples were weighed (W_ο_) and impregnated in distilled water at room temperature. The samples were removed from water gently wiped in tissue paper to soak wateron surface and weighted (W_S_) each hour .The experiment was carried out until 24 h. The swelling was calculated using the following Eq. (1)

1 $${\text{Swelling}}\;\left( \% \right) = \left[ {\left( {{\text{W}}_{{\text{s}}} - {\text{W}}_{{\text{o}}} } \right){\text{/W}}_{{\text{o}}} } \right] \times 100$$

#### FT/IR analysis

Fourier- transform infrared spectra photometer (FT/IR) (Bruker Tensor 37 , Thermo Fisher , USA) with an attenuated total reflection(ATR)was used to record the scans of hydrogels between 4000 and 400 cm^-1^ at resolution of 4 cm^-1^.

#### Mechanical testing

The mechanical properties of the PVA/XG blend films were evaluated using dumbbell-shaped specimens prepared according to ISO 527–2 and ASTM D412a-98 standards. Tensile tests were conducted using a Qchida computerized universal testing machine (Dongguan Haida Equipment Co. Ltd., China) at a crosshead speed of 10 mm/min and a temperature of 25 ± 2 °C. The tensile strength and elongation at break were determined, and the reported values represent the average of three independent measurements for each sample.

#### Scanning electron microscope (SEM)

The fracture surface morphological structure of the samples was studied using a scanning electron microscope (SEM) with a JEOLJSM-5400, Tokyo, Japan. Samples were prepared for the SEM micrographs by fracturing the sample in liquid nitrogen.

#### X-ray diffraction (XRD)

X-ray diffractometer (Shimadzu XRD-6000), XRD patterns were captured. On the diffractometer using a CuK radiation source with a wavelength of 0.1546 nm, a scan rate of 2θ/min was used to obtain XRD patterns. The generator’s output had a 40 kV and 40 mA voltage and current, respectively.

#### Antimicrobial activities

Antimicrobial activities were evaluated against Staphylococcus aurous and salmonella as bacterial activity study. The disk diffusion test was applied to evaluate the antimicrobial activity of composites, which is the most used technique for antimicrobial susceptibility testing This assay is based on the susceptibility of these films to bacterial growth.

#### Fish spoilage

Colored blend samples were placed in separate individual PET boxes and inserted under the opening well. Each box was sealed with a permanent gas-tight seal to prevent leakage of amines. The sensors responses were monitored with the optical scanner described previously^19^. In this paper, blend sensors displayed a color change from brown to dark violet, easily visible to the naked eye. The fish used in all experiments are not live or catched fish but, purchased from the global markets.

### Statistical analysis

All mechanical and swelling measurements were performed in triplicate, and the results are presented as the mean ± standard deviation (SD). Statistical analysis was conducted using one-way analysis of variance (ANOVA), with a significance threshold set at *p* < 0.05. This ensured that observed differences between irradiated and non-irradiated samples were statistically significant and reproducible.

## Results and discussion

**Scheme 1:**
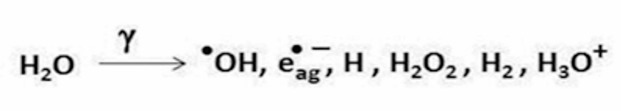
Expected radiolysis species produced by EB-irradiation of water solvent.

Irradiation of polymeric solutions produces numerous species from polymer chains and/or from the water radiolysis during irradiation process. These species, lead to chemical and physical changes that occur in polymer matrix^20^, these species can be formed on polymeric chains directly or/and firstly on solvent molecules as shown in and transferred to polymeric chains (scheme 1). Degradation/crosslinking in polymers can be formed from radicals occur on polymeric chains directly, also can formed from radicals indirectly formed as reported by Charlesby et al.^21,22^ Formed Degradation/crosslinking affecting the properties of polymer blends depending on what process is predominant.

### Swelling properties

**Fig. 1:**
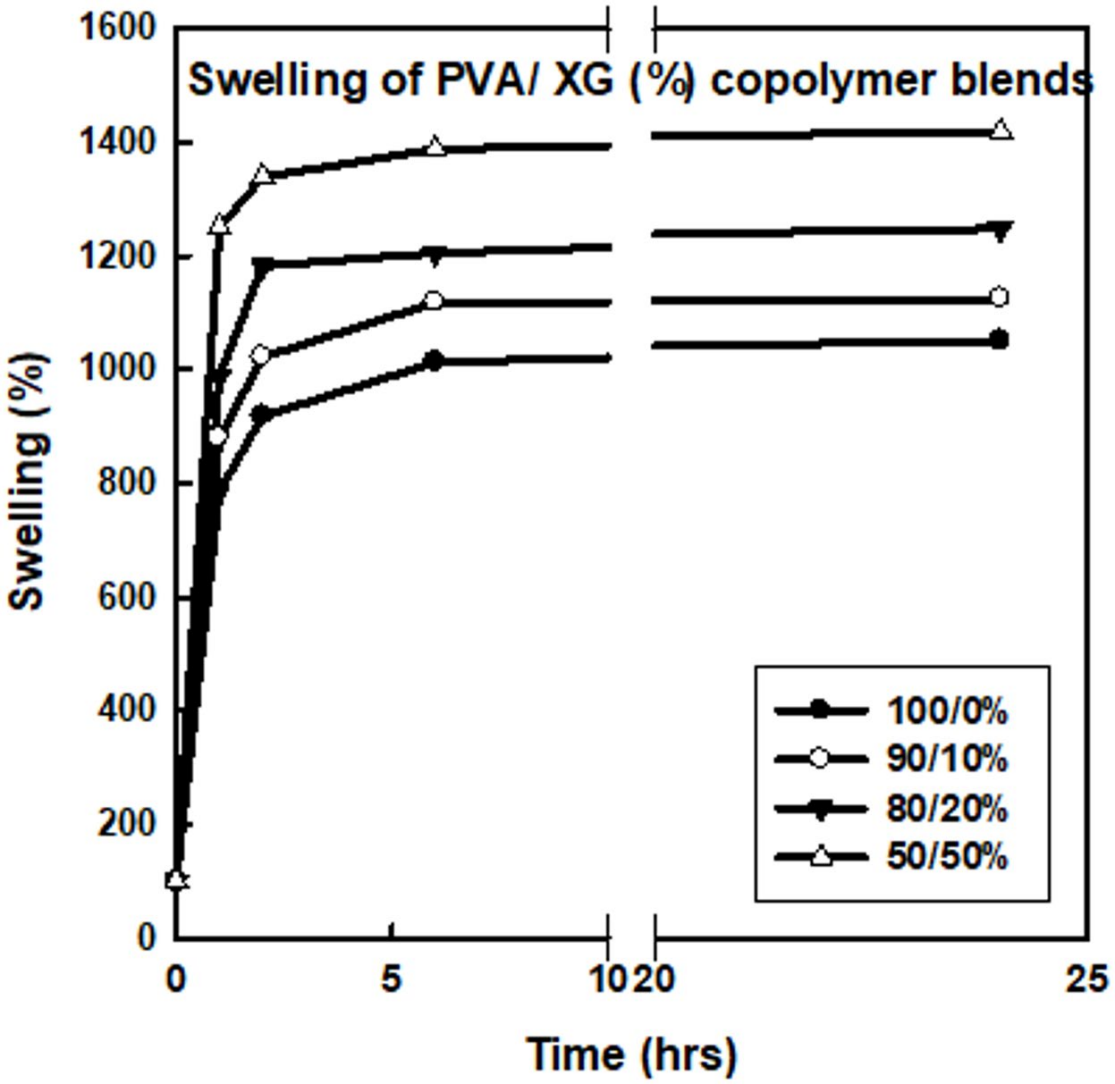
Swelling behavior of PVA and PVA/XG (%) blends of different compositions EB-irradiated to a dose of 15 kGy.

Figure 1 displays the swelling behavior of pure PVA and PVA/XG polymer blends at various ratios, after EB-irradiated to 15 kGy. It can be seen that the amount of swelling was reached a maximum values after (2–7) hours depending on the polymer blend composition. However, further increase in swelling time up to 24 h, showed no further increases in the swelling values. Meanwhile, it can be noticed that the swelling properties depend largely on the blend ratios and increased with increasing the XG ratio. In addition, the swelling values have shown a maximum value with increasing the XG ratio in the blend. The formed copolymer blend is a semi-interpenetrating network hydrogel, in which the XG is engaged in cross-linked PVA network though hydrogen bonding as in scheme 1^23^. The formation of a semi-interpenetrating polymer network (semi-IPN) between PVA and XG under EB irradiation indeed restricts the swelling capacity of the films due to the establishment of intermolecular hydrogen bonding and partial crosslinking between the two components. The hydrogen-bonded network limits the diffusion of water molecules into the polymer matrix by reducing the number of free hydroxyl groups available for hydration. However, a moderate degree of blending (≤ 30 wt% XG) or irradiation (≤ 15 kGy) results in an optimal balance between structural integrity and water absorption, as the XG chains introduce additional hydrophilic sites that counteract excessive densification of the network. This explains the observed non-linear swelling trend, where swelling slightly decreases at higher doses or XG contents due to network densification and reduced free volume. Similar results have been reported for other PVA/polysaccharide semi-IPN hydrogels, where inter-chain bonding enhances dimensional stability but limits water uptake beyond a critical crosslinking density^15^.

### FT/IR spectroscopy

**Fig. 2:**
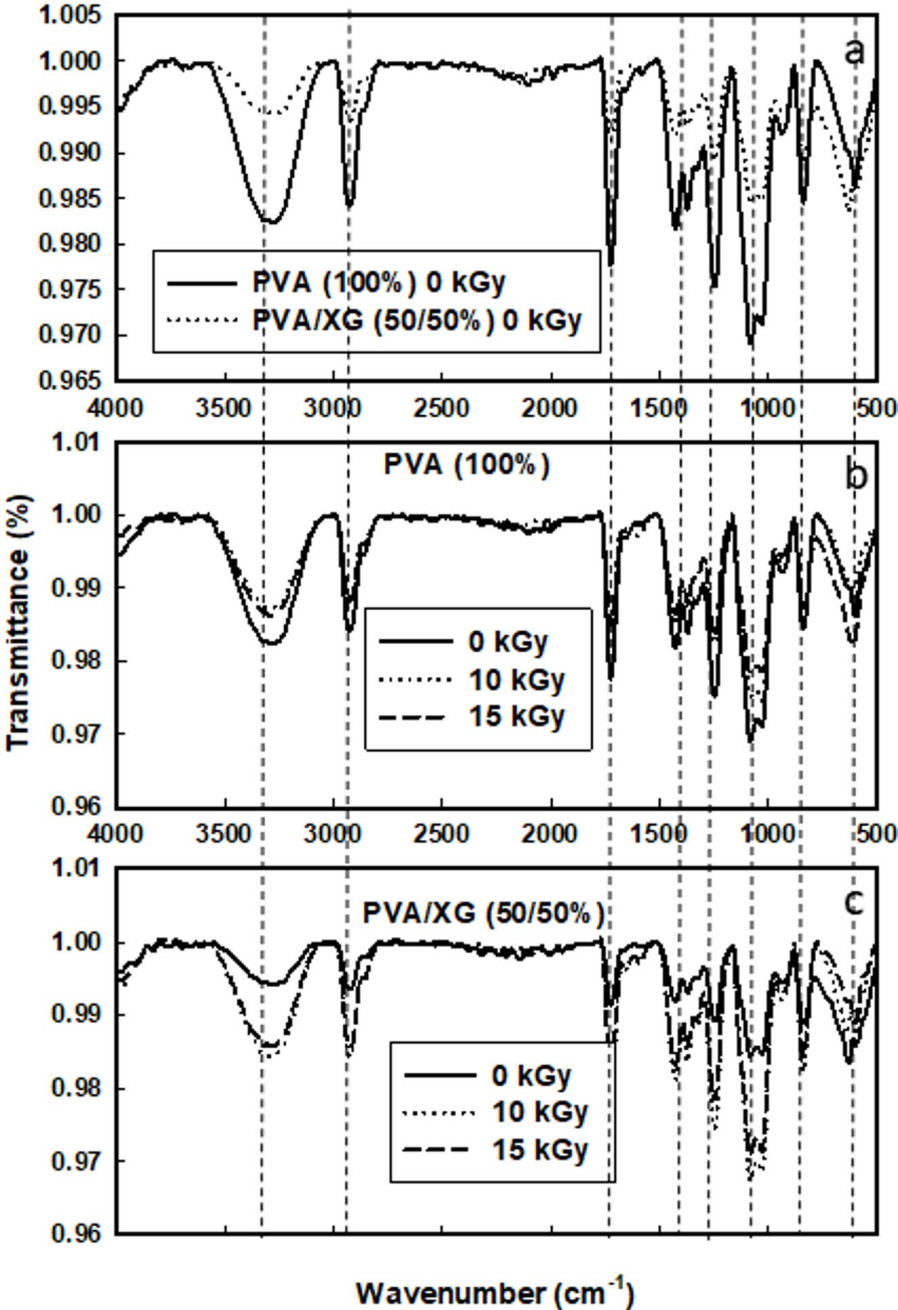
FT-IR spectra of PVA (100%) and PVA/XG (50/50%) copolymer blends, before and after they had been EB- irradiation to various doses.

FTIR spectroscopy is the tool employed to investigate the chemical changes/interactions that can be induced by EB irradiation in between the polymer blends components. The FTIR spectroscopy of pure PVA and PVA/XG (50/50%) were shown in Fig. 2a–c. As shown in Fig. 2a, the characteristic peaks PVA such as 3355 and 2931 cm^-1^ that attributed to the stretching vibration of O–H and C-H, respectively. The peaks at 1432, 1054, 946 and 842 cm^-1^ are attributed to C-H in –(CH_2_) wagging, C-O, CH_2_ and C–C stretching vibration, respectfully. On the other hand, the FT/IR spectra of XG showed characteristic absorption bands at 1425 and 642 cm^-1^ due to presence of (C-O–O) groups; (C-O) asymmetric and symmetric stretching vibration. Other bonds appeared at 2939, 1200–990 cm^-1^ that are common to all polysaccharides due to O–H and C-H bonds in –CH_2_ groups located on glucose rings^24^. For PVA/XG blends, the FT/IR spectra could confirm the characteristic absorption peaks of both PVA and XG indicating their contribution in obtained blends. In the absorption region of 3000–3600 cm^-1^ that due to the OH groups stretching vibration which appeared in the PVA at 3331 cm^-1^ were shifted to lower frequency, when XG (OH = 3280 cm^-1^) was blended to PVA. This is due to the interaction between PVA chains and XG chains^25^. Also, the region at 1815–1630 cm^-1^, which due to the C = O groups stretching for XG appeared at 1735 cm^-1^ and PVA at 1644 cm^-1^, peaks 1088 cm^-1^ is shifted to 1077 cm^-1^ due to hydrogen bonding between primary –OH and secondary (-OH)^8,26^. Figure 2c shows the effect of EB-irradiation on PVA or PVA/XG (50/50%) It can be seen that the characteristic bands for irradiated PVA are decreased in band intensity and this due to the involvement of these groups in crosslinking process that occur within this range of EB-irradiation doses. On the other hand, Fig. 2c Showed that the irradiation after addition of XG (50%) to PVA the characteristic bands were increased in its intensities and that due to oxidative degradation occur on XG . Usually, the final effect is combination between the effects that occur on blend components (PVA crosslinking and XG degradation) at this range of irradiation doses. The observed spectral variations in the FT/IR spectra of the irradiated PVA/XG blends can be clearly attributed to two competing mechanisms crosslinking and degradation depending on the irradiation dose. The broad band around 3355–3280 cm⁻^1^ corresponding to O–H stretching vibrations exhibits a noticeable reduction in intensity after irradiation, which indicates the formation of new hydrogen bonds and partial consumption of hydroxyl groups, confirming the occurrence of intermolecular crosslinking within the PVA chains^27^. Meanwhile, the emerging absorption bands near 1735 cm⁻^1^ and 1644 cm⁻^1^ are related to carbonyl (C = O) and C = C stretching vibrations, respectively, which become more prominent at higher irradiation doses. These bands arise from oxidative degradation of the XG backbone, leading to chain scission and formation of aldehyde and carboxylic end groups^28^. The differentiation between these two processes is therefore dose-dependent: at low to moderate doses (≤ 15 kGy), crosslinking dominates due to radical recombination within PVA segments; whereas at higher doses (≥ 20 kGy), oxidative degradation of polysaccharide components becomes more evident. This interpretation is consistent with previous radiation-induced changes observed in other PVA-based blends^29,30^.

### Mechanical properties

**Fig. 3:**
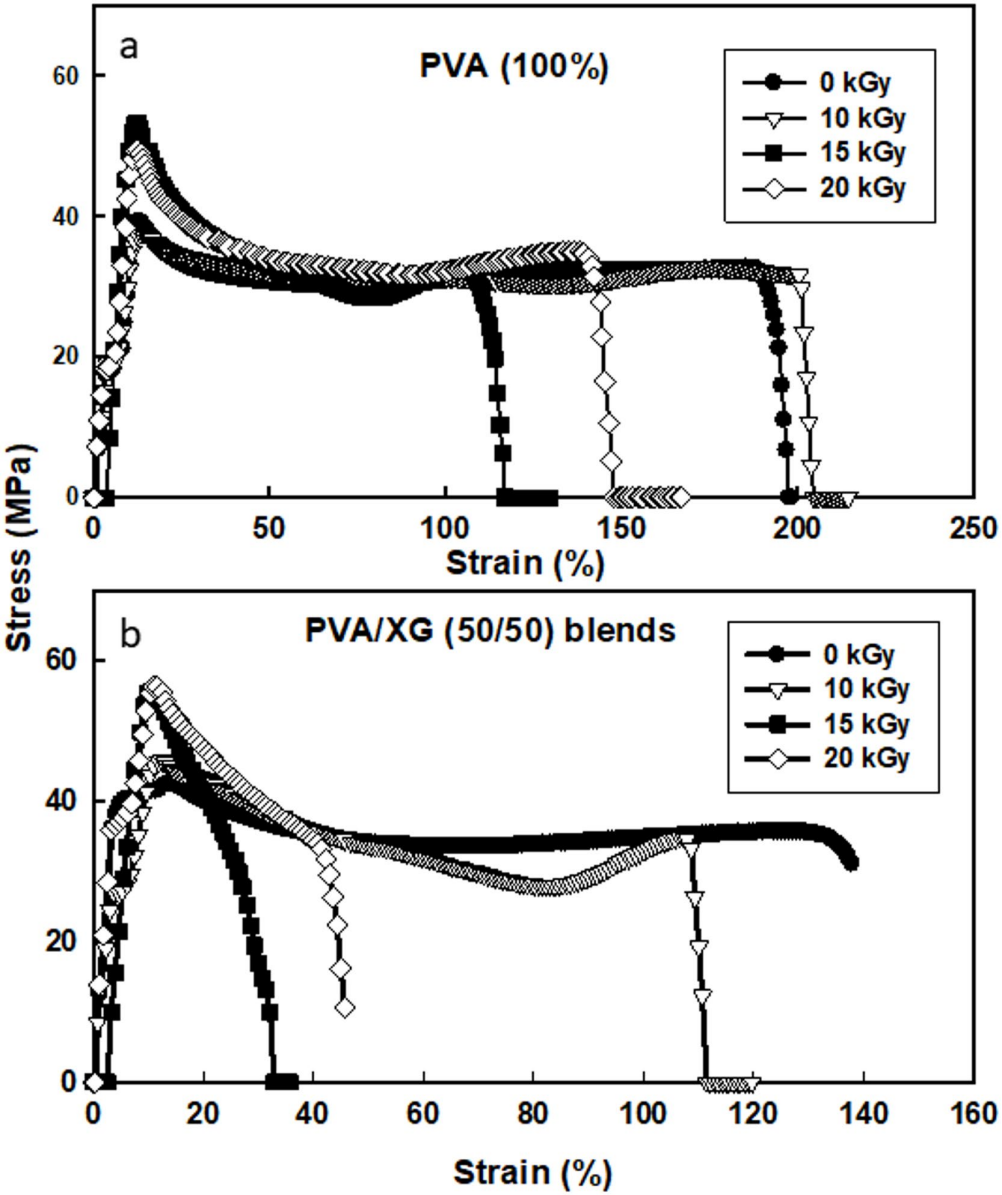
Stress–strain curves of unirradiated and EB-irradiated of PVA (100%) and PVA/XG (50/50%) copolymer blend to various doses.

Mechanical properties of polymeric materials are important parameter for different packaging applications. Stress–strain curves of polymeric materials can be divided into three types reflecting the polymeric structure and its mechanical properties. The first in hard polymeric materials (i.e. Polystyrene) the stress property is increased rapidly with little strain (%) and then breaks. The second type in tough polymeric materials (i.e. Polyethylene), in which the stress property is increased with little increase in strain percentage until yielding point then followed by cold drawing and large increase in strain % without increase in stress until breaking point. The last type in blastomeric materials (i.e. rubber) the strain percentage increases with little increase in stress property until break point. In our polymer blends, the stress/strain curves for un-irradiated and EB-irradiated PVA/XG (50/50%) copolymer blends are shown in Fig. 3. From these Figure, few pints may be addressed:

1. The stress–strain curves are similar to tough polymers with yielding and drawing before breaking. Also, EB- irradiation improves the tensile property of PVA with decreasing in strain percentage property without change of the shape of the stress–strain curves. This effect was expected due to EB-irradiation crosslinking for PVA chains.
2. The incorporation of XG to PVA (50% wt) increases the tensile properties with decreasing in the strain (%) but without change in the shape of stress–strain curve. This behavior is due to phase separation that can be occurred between blend components at this ratio (50/50%). SEM micrographs (in the next section, Fig. 6) support this assumption by displaying distinct morphological domains corresponding to PVA-rich and XG-rich phases. The smoother, compact surfaces observed at 10 kGy indicate improved miscibility and interfacial adhesion between the two polymers, while higher XG loading (≥ 50 wt %) led to visible heterogeneous patterns, confirming partial phase separation. Similar findings have been reported by Zhang et al.^31^, where polymer blends containing polysaccharides exhibited dual-phase morphologies responsible for increased tensile strength but reduced flexibility.
3. EB- irradiation of PVA/XG (50/50%) increases the tensile properties with decrease in strain property. In addition, the curves shape were goes to hard type stress–strain shape.

**Fig. 4:**
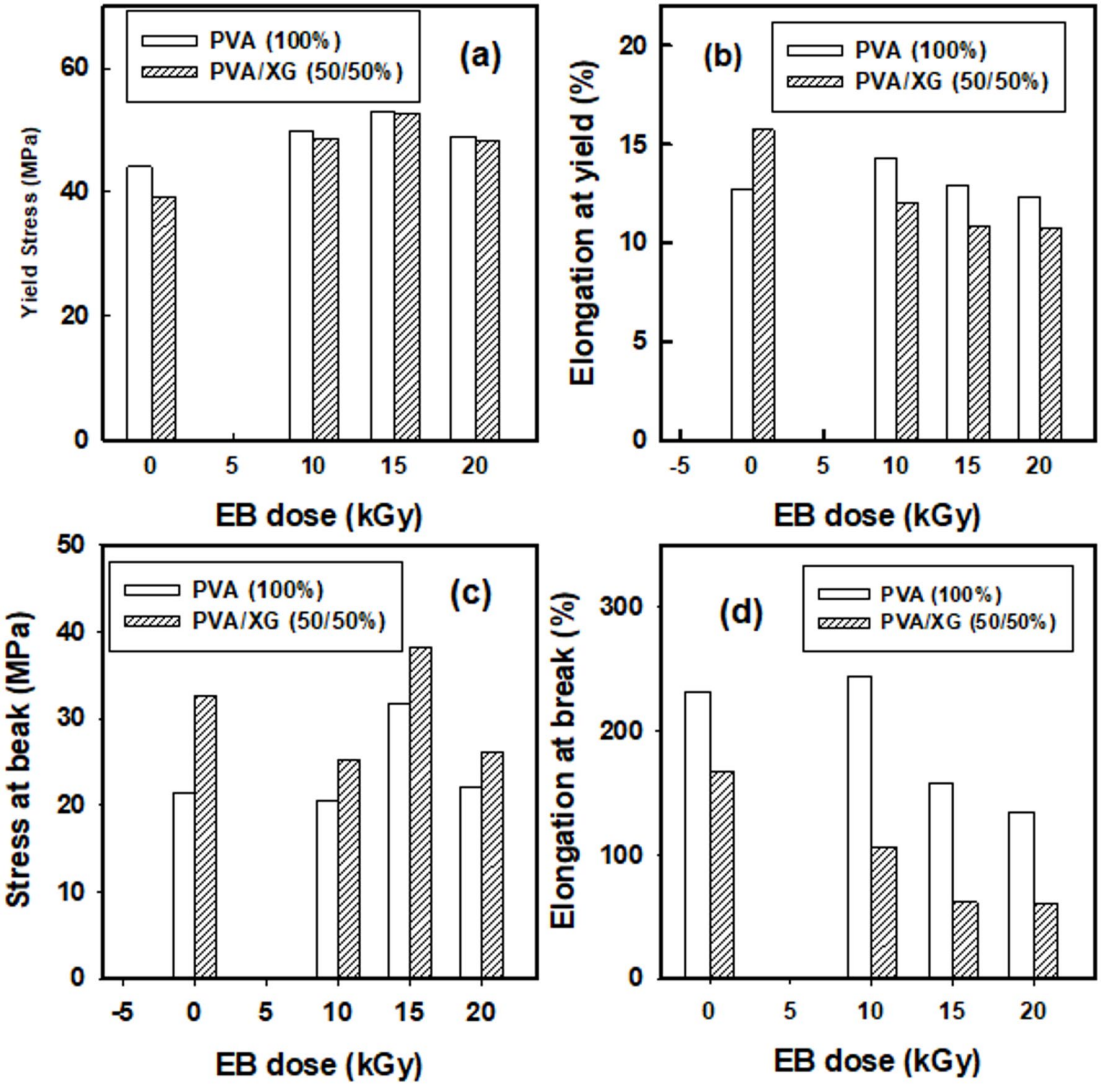
Tensile mechanical parameters of unirradiated and EB-irradiated PVA (100%) and PVA/XG (50/50%) copolymer blends to various doses.

Figure 4a–d shows the tensile and elongation values at yield/break points for PVA as well as PVA/XG (50/50%) blends EB-irradiated to various doses. As shown in Fig. 4a, b, the tensile properties at yielding point were increased for (PVA 100%) from 39 to 53 MPa upon EB- irradiation to a dose of 15 kGy , Also , the break tensile properties were increased from 21 to 32 MPa upon EB–irradiation to 15 kGy. In Fig. 4c, d, the elongation (%) was constant at yield point (13–12) % with EB- irradiation up to 20 kGy. Also, the elongation % was decreased with increasing irradiation doses in PVA/XG (50/50%) from these figures we can be attributed to this behavior is that the XG is more brittle and harder than PVA so the addition of XG to PVA films increase the tensile from ( 39 to 44 MPa) with decrease in elongation % (232 to 134%). EB-irradiation can be affect on the polymer blend through cross-linking process that occur in PVA at this range of irradiation doses, whereas the XG is radiation degradable polymer as most polysaccharides. From these findings, some points can be addressed:

1. Tensile values of PVA at yielding/breaking points were affected by EB-irradiation and by adding 50% XG. This effect is clear in yielding point than breaking point.
2. The strain % values for PVA at yielding points were not affected clearly by irradiation or by adding XG. On the other hand the values at break points were affected by adding XG or by irradiation.
3. Addition of XG to PVA at this concentration have some sort of phase separation that decreases the strain properties, but EB-irradiation of these blends induces crosslinks between PVA chains that can be hold the degraded parts of XG chains.

The enhancement in tensile strength of the irradiated PVA/XG blends is mainly attributed to crosslinking within the PVA component rather than chain scission of the xanthan gum chains. Chain scission of XG typically results in molecular weight reduction and mechanical weakening, which would cause a drop rather than an increase in tensile strength. In contrast, the EB irradiation process promotes the formation of inter and intramolecular crosslinks within PVA chains through radical recombination, generating a three-dimensional polymeric network that improves structural rigidity and load transfer efficiency^14^. Additionally, the stable elongation values observed up to 15 kGy, along with improved tensile modulus, confirm that the matrix strengthening results from crosslinked PVA domains instead of degradation-induced hardening. Similar conclusions were drawn by Liang et al.^32^, who reported that moderate electron beam irradiation enhances tensile properties of PVA-based systems by network reinforcement, while excessive doses lead to chain scission and embrittlement. Hence, the mechanical reinforcement observed in this study can be confidently ascribed to the predominance of PVA crosslinking over XG degradation at moderate irradiation doses^33^.

### Scanning electron microscopy

**Fig. 5:**
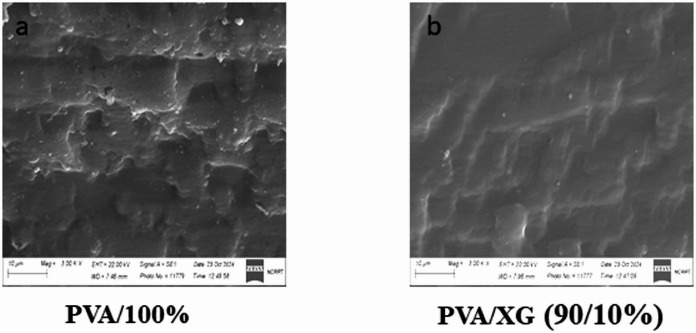
SEM micrographs of the fracture surface morphology of PVA/XG (%) copolymer blends EB- irradiated to a dose of 25 kGy.

SEM technique is one of common tools for imaging the structure surface morphology and its microstructure for polymeric materials. The SEM micrographs of 25 kGy irradiated PVA containing different ratios of XG is shown in Fig. 5. It can be seen that the micrograph of PVA (100%–25 kGy) is smooth without any cracking (i.e. morphology without any air bubble or rapture)^34^. Addition of XG to PVA showed some layers that increase with increasing the XG %. This behavior may be due to that the blend containing two polymers with different behavior under EB- irradiation; in which XG is degradable polymer whereas the PVA is crosslinking polymer.

**Fig. 6:**
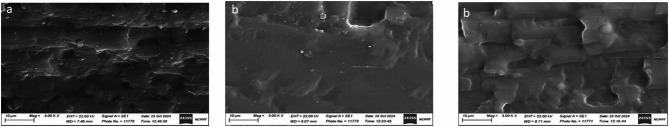
SEM micrograph of the fracture surface morphology of PVA/XG (50/50) copolymer blends EB-irradiated with varies doses.

Figure 6 shows the SEM micrographs of the fracture surface of PVA/XG (50/50%) copolymer blends EB-irradiated to various doses. The fracture surface of the sample electron beam irradiated to 10 kGy showed uniform dispersion of XG in PVA at this concentration, with some layered and pores as evidenced by SEM micrographs. These layers were increased with increasing EB- irradiation doses to 15 kGy that may be due to PVA cross-linking with increasing PVA cross-linking^35^.

### XRD analysis

**Fig. 7:**
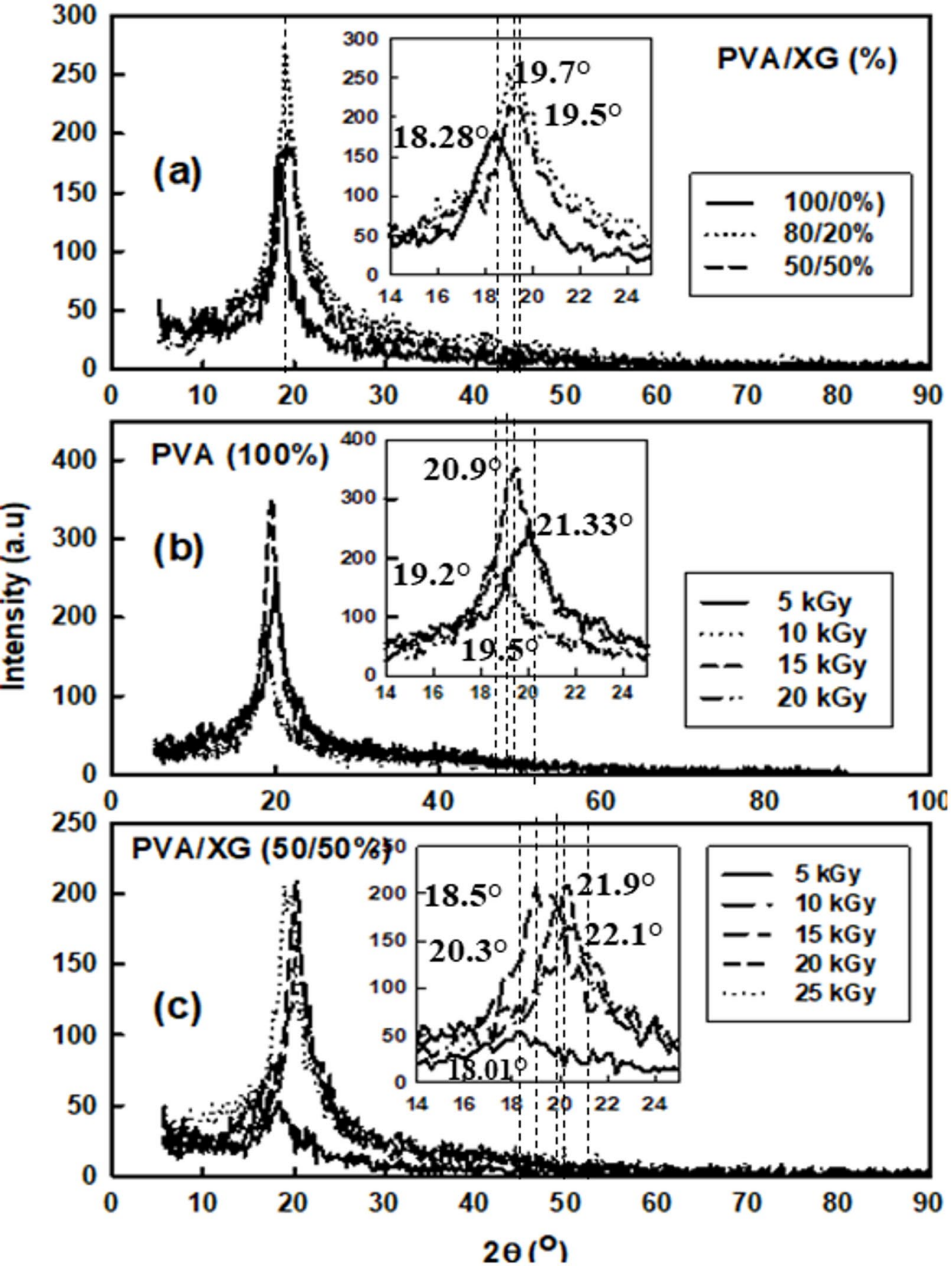
XRD patterns of PVA and PVA/XG (50/50) copolymer blends, before and after they had been EB-irradiated to various doses.

The XRD patterns were used to investigate the crystallinity structure changes occurred in PVA due to blending with XG and the EB irradiation the XRD pattern for PVA containing different ratios of XG was shown in Fig. 7a–c. Also, irradiated PVA and PVA/XG (50/50%) to various doses were shown in Fig. 7b, c. Basically, XG has an amorphous structure and exhibit a broad peak at about 2ϴ = 19°; results from the intermolecular hydrogen bonding between –OH groups. PVA polymers, however has a semi-crystalline structure that exhibit a peak around 2ϴ = 20^36^. In addition, of XG to PVA at different ratios has a little shift in 2ϴ to higher position that may be due to presence of PVA and XG together in solution are forming straight chains that alleged together by hydrogen bonding and forming layers in drying. The effect of EB- irradiation of PVA and PVA/XG (50/50%) at various doses is shown in Fig. 7b, c. It can be seen that the EB- irradiation to a dose of15 kGy) of PVA solution has a little shift to lower 2ϴ that with increasing in the peak intensity, this shift may be due to EB- irradiation crosslinking between chain that restricted the semi-crystalline formation on drying. Zhang, Q, et al. Found that PVA/XG prepared by freezing/throwing process, the crosslinks between PVA/XG facilitated the formation of crystal nucleus in PVA^8^. The addition of XG to PVA with 50% and EB-irradiation, the pattern is changed whereas XG is non-crystalline polymer that mixed with semi-crystalline polymer (PVA), the peak intensity decreased, with irradiation to (15–20) kGy the peaks were shifted to higher 2ϴ (~ 20.4) and then decreased to lower 2ϴ (19.5o). With EB- irradiation to a dose 25 kGy, the decrease may be due to the crosslinks between PVA chains restricted crystallization of PVA. The effect of electron beam (EB) irradiation on the crystallinity of PVA/XG blends exhibits a non-linear behavior that depends strongly on the irradiation dose. At moderate doses (≤ 15 kGy), the XRD patterns show a noticeable increase in the intensity of the main diffraction peak near 2θ = 19.6°, corresponding to the (101) plane of semi-crystalline PVA. This enhancement indicates partial ordering and alignment of polymer chains caused by radiation-induced crosslinking, which restricts molecular mobility and promotes microcrystalline domain formation within the amorphous matrix. However, when the irradiation dose exceeds 20–25 kGy, a significant reduction in diffraction intensity and peak sharpness is observed, signifying a loss of crystalline order. This decrease results from excessive crosslinking and chain scission, which disturb the periodic packing of PVA chains and enhance amorphous character. Such a two-stage behavior initial crystallinity enhancement followed by suppression at higher doses has also been documented in other EB-irradiated PVA-based systems^14,37^. Therefore, it can be concluded that EB irradiation initially enhances crystallinity through network ordering and hydrogen bond rearrangement, but prolonged exposure or higher doses induce structural damage and amorphization due to excessive radical attack.

## Application of prepared blends as a freshness indicator in food packaging

After food packaging, the gas composition within the package is changed due to the activity of the food, the nature of package material, and the environmental conditions (i.e. gas generation by microorganisms , gas transition through package material or package lacks)^38^. Gas indicators are small materials that form as package label and responded to gas composition changes and helping in monitoring the quality and safety of packaged food products. The prepared polymer blends were investigated for monitoring the fish spoilage through the color change that may occur due to gas generation by microorganisms. In this regard the films were tested to susceptibility to microbial agents, and the color changes occur due to fish spoilage (NH_4_OH evolving) was investigated^39^.

### Susceptibility to microbial contamination

**Fig. 8:**
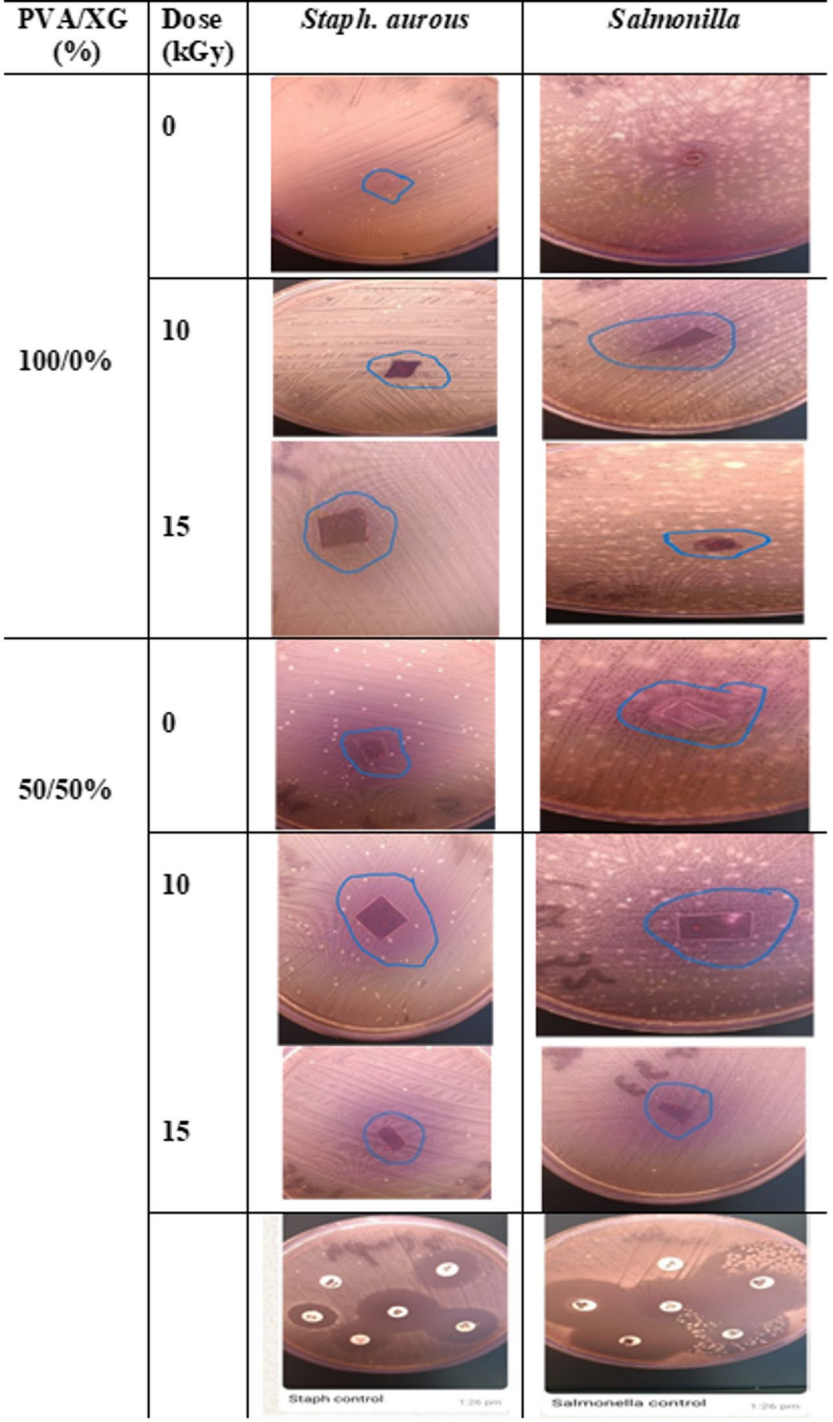
Antimicrobial activity, evaluated by inhibition zone, of PVA and PVA/XG (50/50) copolymer blends, before and after EB-irradiation to various doses.

The susceptibility of prepared blends to microbial contamination is an important factor that can prevent the food contamination. Antimicrobial test were done to identify which are effective against surface growth of microorganisms. Discs for prepared blends were subjected to both S. aureus and Salmonella bacteria. The obtained results are shown in Fig. 8. From Fig. 8 it can be seen that the un-irradiated PVA and PVA/XG (50/50%) were susceptible to both S. aureus and Salmonella bacteria. On other hands, the EB-irradiated samples were not susceptible to bacteria growth, also, 10 kGy-irradiated samples was suitable to give a blend films with resistivity to S. aureus and Salmonella bacteria susceptibility.

### Freshness indicator property

**Fig. 9:**
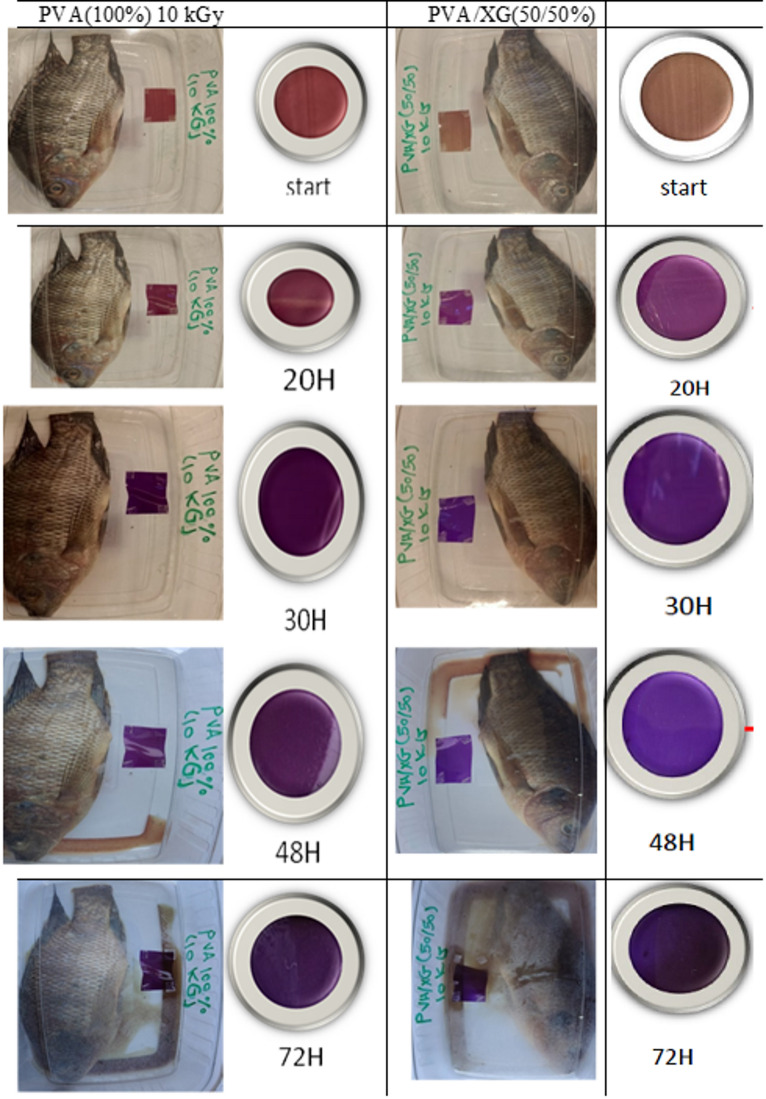
Images of Fish spoilage during 72 h of the PVA and PVA/XG (50/50%) copolymer blends, EB-irradiated to a dose of 10 kGy.

**Fig. 10:**
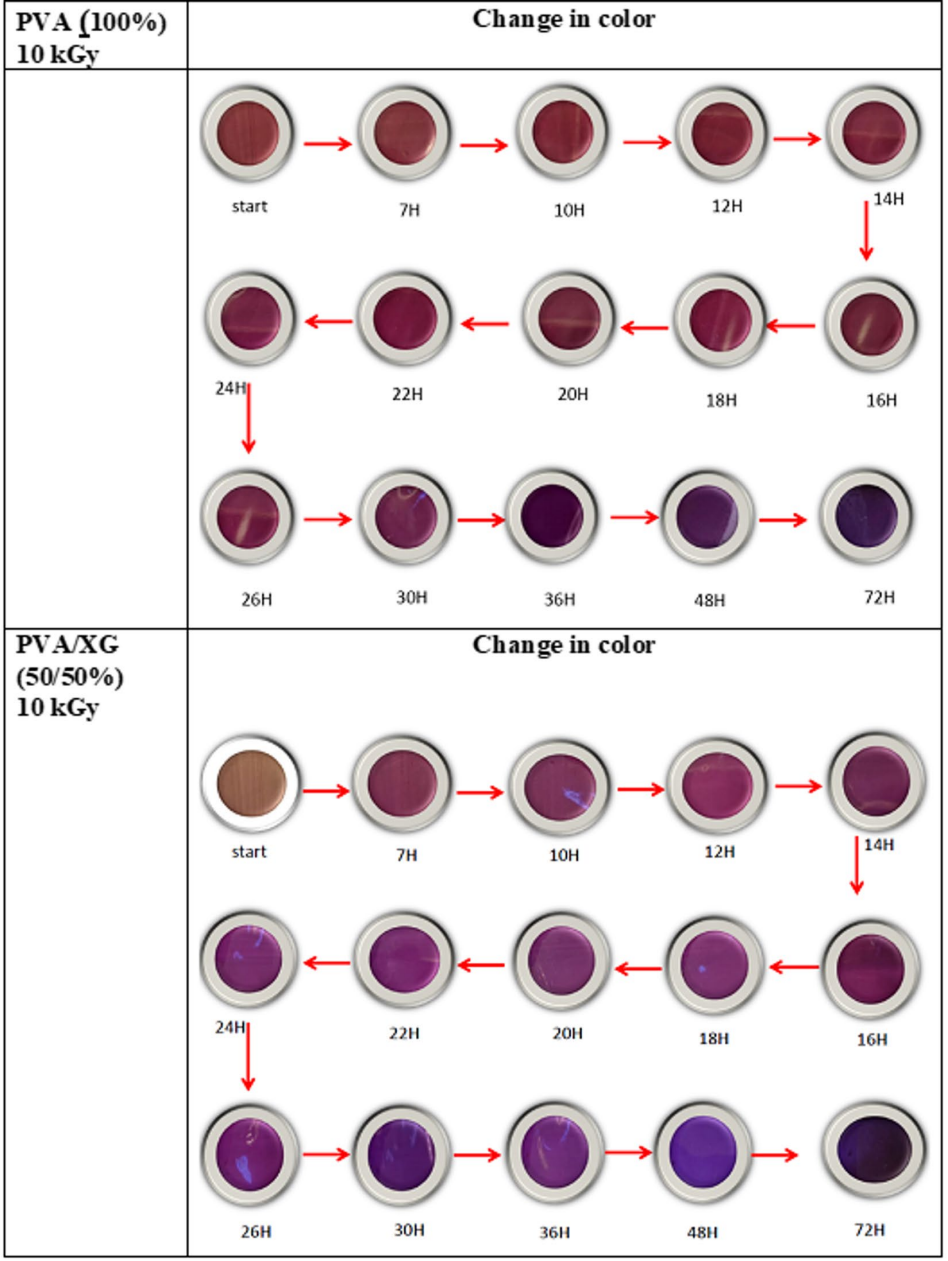
Hourly color change in PVA and PVA/XG (50/50%) that EB-irradiated to a dose of (10 kGy).

The information provided by packaging system on the quality of food product may be either indirect (change in O_2_ concentration) or directly. Freshness indicators imply product quality information resulting from microbial growth or chemical changes within food products. They can be developed depending on the base of metabolites associated with microbiologically induced deterioration. Biogenic amines have been connected as indicators for meat products decomposition^40^. The effeteness of amine indicators was a goal of our prepared polymer blends. The freshness indicator of polymer blend based on un-irradiated and 10 kGy EB-irradiated PVA and PVA/XG (50/50%) are shown in Fig. 9. it can be seen that the color was clear changed from brown to dark violet by fish spoilage during 3 day storage at room temperature. In addition, the hourly change in the color was shown in Fig. 9 and the hourly change in the color was shown in Fig. 10. The response of EB-irradiated PVA/XG (50/50%) blend to fish spoilage was clearly than that in irradiated PVA and this may be that presence of XG in the matrix improve the absorption of ammonia gases.

From microbial susceptibility and freshness indicator experiments we can conclude that blends containing 50% of its composition natural polymer (XG) and irradiated to (10-15 kGy) can be used an easy color indicator about the freshness of fish without susceptibility to microorganisms.

## Conclusion

Electron beam (EB) irradiation was successfully utilized to tailor the structural, mechanical, and sensing properties of PVA/XG blend films for potential application in biodegradable food packaging. The results revealed that moderate irradiation doses (10–15 kGy) significantly enhanced the tensile strength from 18.6 MPa (neat PVA) to 28.4 MPa (PVA/XG 50/50 at 10 kGy), accompanied by a controlled reduction in elongation, indicating improved crosslink density and inter-chain hydrogen bonding. FT/IR analysis confirmed radiation-induced structural rearrangements through the appearance of stronger C = O and O–H interactions, while XRD patterns showed a temporary increase in crystallinity at 15 kGy, followed by partial amorphization at higher doses. SEM images demonstrated uniform dispersion and partial phase separation at higher XG loadings, consistent with the observed mechanical trends. In addition, the irradiated films exhibited enhanced gas sensitivity toward volatile amines released from decomposing fish, particularly at 10–15 kGy, confirming their suitability as smart indicators for monitoring food freshness. Overall, this work highlights a simple, additive-free, and tunable strategy for developing biodegradable polymeric films with dual functionality, improved mechanical stability and real-time gas-sensing capability through controlled EB irradiation.

## Data Availability

The data and materials of this study are declared to be available by the authors. Interested parties can request for the datasets generated during the study from the corresponding author (Hussein Oraby Hussein.mohamed4544@yahoo.com) on reasonable.
